# Supplementary material for: WordCommentsAnalyzer: A windows software tool for qualitative research
Source: F1000Res. 2018 Sep 4;7:536. Originally published 2018 May 3. [Version 2] doi: 10.12688/f1000research.14819.2 (PMC6137411; doi:10.12688/f1000research.14819.2)
Supplement: Supplementary file 3 [file f1000research-7-17614-s0002.tgz › b2a79496-4ada-47c4-9682-a99ce867836d.zip!/Eight Iranian Health Journals/The Journal of Toloo-e-Behdasht_30.docx]

© The Journal of Toloo-e-Behdasht. This work is licensed under CC BY 4.0 (https://creativecommons.org/licenses/by/4.0/).

Volume & Issue: دوره 12 - شماره 2

Title: بررسی مقایسه ای روش آموزشی کودک به کودک و مربی به کودک بر اساس مدل اعتقادبهداشتی در ارتقاء بهداشت دهان و دندان دانش آموزان مدارس پسرانه ابتدایی شهر سنندج

http://tbj.ssu.ac.ir/article-1-553-fa.html

مقدمه : مشکلات بهداشت دهان و دندان یکی از مهم ترین مشکلات بهداشتی شایع در سنین مدرسه می باشد. متاسفانه روش های رایج آموزش بهداشت در مدارس در زمینه کنترل این مشکلات موفقیت زیادی نداشته اند. لذا با توجه به اهمیت موضوع پژوهش حاضر با هدف تعیین میزان کارآیی روش آموزشی کودک به کودک در مقایسه با روش مربی به کودک بر مبنای مدل اعتقاد بهداشتی به منظور ارتقاء بهداشت دهان و دندان در دانش آموزان مدارس پسرانه ابتدایی شهر سنندج انجام شده است.   روش بررسی : این پژوهش یک مطالعه مداخله ای نیمه تجربی می باشد. بدین منظور سه مدرسه از میان مدارس پسرانه ابتدایی شهر سنندج به روش تصادفی انتخاب گردیدند و سپس یک مدرسه به عنوان گروه کودک به کودک (30 نفر ) و یک مدرسه به عنوان گروه مربی به کودک (30 نفر) و یک مدرسه نیز به عنوان گروه شاهد (30 نفر) در نظر گرفته شد. شرکت کنندگان قبل و 2 ماه پس از انجام مداخله آموزشی با تکمیل پرسشنامه مورد بررسی قرار گرفتند. پس از انجام ارزشیابی تشخیصی، مداخله آموزشی بر اساس مدل اعتقادبهداشتی در زمینه آگاهی از بهداشت دهان و دندان و نحوه استفاده از مسواک در بین دانش آموزان صورت گرفت. استراتژی اصلی ارتباطی بکار رفته در پژوهش استفاده از گروه همسالان بود.   یافته ها: نتایج بدست آمده نشان می دهد که بعد از انجام مداخله آموزشی گروه کودک به کودک اختلاف معنی داری در زمینه نمره آگاهی (726/0= P )، حساسیت درک شده (785/0 = P )، منافع در ک شده (898/0= P )، موانع درک شده (95/0= P- ) وراهنمای عمل (95/0= P ) با گروه مربی به کودک ندارد. اما در زمینه شدت درک شده (002/0= P ) و بهبود عملکرد(افزایش استفاده ازمسواک) (007/0= P ) بهتر عمل نموده است.   نتیجه گیر ی:نتایج این پژوهش نشان می دهدکه در زمینه ارتقاء بهداشت دهان و دندان و افزایش استفاده از مسواک، گروه کودک به کودک با عملکردی نزدیک به گروه مربی به کودک موفقیت چشمگیری داشته است بنا بر این با توجه به توان بالقوه دانش آموزان در آموزش همسالان، استفاده از رویکرد آموزشی کودک به کودک در برنامه های آموزش بهداشت توصیه می شود.

Title: کارایی جاذب ساقه جو در حذف یون کادمیوم از محلول‌های آبی باروش جریان پیوسته

http://tbj.ssu.ac.ir/article-1-1811-fa.html

مقدمه: انتشار فلزات سنگین در محیط زیست ناشی از صنعتی شدن و شهرنشینی، یک مسئله نگران کننده در سراسر جهان می‌باشد. یون کادمیوم یکی از فلزات سنگین غیر ضروری، غیر مفید و خیلی سمی برای انسان، حیوانات و گیاهان است که حداکثر غلظت مجاز آن 005/0 میلی گرم در لیتر توسطWHO تعیین شده است. روش‌های مختلفی برای حذف فلزات سنگین مطالعه شده اما استفاده از جاذب‌های ارزان قیمت برای حذف این فلزات در سال‌های اخیر مورد توجه پژوهشگران قرار گرفته است. در این تحقیق روش جریان پیوسته را برای حذف یون کادمیوم از محلول‌های آبی توسط جاذب تهیه شده از ساقه جو مطالعه شد. روش‌بررسی : این مطالعه تجربی ـ آزمایشگاهی در سال 1390 انجام شد. ذرات ساقه جو در محدوده 19/1 تا 2 میلیمتر در ستونی با جریان روبه پایین برای حذف یون کادمیوم مورد بررسی قرار گرفت. در این بررسی pH برابر 6 ودمایC°27 آزمایش‌ها ثابت بود واثر متغیرهای غلظت اولیه ورودی، دبی جریان ورودی و ارتفاع بستر جاذب بر میزان حذف یون کادمیوم مطالعه شد. برای هر آزمایش مقدار 6 میلی لیتر نمونه در زمان‌های 30 دقیقه از خروجی ستون جمع آوری و غلظت یون کادمیوم در هر نمونه با دستگاه جذب اتمی اندازه گیری شد. یافته‌ها : نتایج آزمایش‌ها نشان داد که منحنی شکست ستون جاذب به متغیرهای مطالعه شده وابسته است. افزایش دبی و غلظت، زمان رسیدن به نقاط شکست و اشباع جاذب راکوتاه‌تر و افزایش عمق بستر آنرا طولانی‌تر می‌کند.  ماکزیمم یون کادمیوم جذب شده بر روی جاذب mg/g07/10در شرایط دبی ml/min 3 ، عمق بستر  cm 30 و غلظت اولیه mg/l 30  بدست آمد. نتیجه‌گیری: با توجه به نتایج بدست آمده، با افزایش دبی ورودی میزان ظرفیت جاذب و راندمان جذب کاهش می‌یابد. در دبی ورودی ml/min 2 یون کادمیوم بخوبی از روی ستون با جاذب ساقه جو حذف می‌شود.داده ها نشان دادند که این جاذب برای جذب یون کادمیوم ظرفیت جذب نسبتاً بالایی دارد. لذا پیشنهاد می‌شود روش جریان پیوسته با پارامترهای بدست آمده در این مطالعه در فرآیند تصفیه شیمیایی آب وفاضلاب مورد بررسی قرار گیرد.

Title: بررسی میزان آلاینده های هوا در محیط پیرامون مجتمع صنعتی بارز

http://tbj.ssu.ac.ir/article-1-555-fa.html

مقدمه: پیامدهای مختلف آلودگی هوا به خصوص اثرات بهداشتی آن از جمله ایجاد و تشدید بیماریهای تنفسی، عصبی، قلبی و عروقی باعث شده است که نظارت وکنترل کیفیت هوا به صورت امری اجتناب ناپذیر در اغلب جوامع شهری و مناطق صنعتی در رأس مسائل ملی مطرح شود . صنایع از عوامل مهم آلاینده محیط می باشند که در مرحله اول، پیرامون خود را آلوده می کنند. از آنجا که تا کنون پایش آلودگی هوا در هوای پیرامون مجتمع صنعتی بارز صورت نگرفته لذا انجام تحقیقی در زمینه بررسی کیفیت هوا ضروری به نظر رسید.   روش بررسی: تحقیق توصیفی- مقطعی است که در بازه زمانی اول خرداد تا پایان آبان ماه 1390انجام گرفت. پایش آلاینده های شاخص این صنعت شامل ذرات (دوده PM10 ) ، هیدروکربورها ( HC )، دی اکسید ازت ( NO2 )،دی اکسید گوگرد( SO2 )،منوکسید کربن( CO )،اوزن( O3 ) و ذرات راسب از طریق درون یابی بین غلظت آلاینده ها در محوطه کارخانه و مقایسه آلاینده ها با رهنمودهای WHO واستاندارد ایران صورت گرفت , هوای پیرامون مجتمع در چهار جهت متقارن و دو شعاع مختلف، در هشت ایستگاه ،ماهیانه سه نوبت به مدت شش ماه مورد اندازه گیری و ارزشیابی قرار گرفت، تعداد 152نمونه از هر آلاینده، جمعا 912 نمونه و 48 نمونه از ذرات راسب برداشته شد .کیفیت بهداشتی هوا بر مبنای استاندارد ایران تعیین گردید.   یافته ها: نتایج بدست آمده از آزمایشات نشان داد که میانگین غلظت بیشینه روزانه ذرات معلق( PM10 ) برابر 65 / 127، دی اکسیدازت( NO2 ) برابر ppb 14/38، دی اکسیدگوگرد( SO2 ) برابر ppb 4/88 ، مونواکسیدکربن( CO ) برابر ppm 38 /1، اوزن ( O3 ) برابر ppb 19/49، هیدروکربورهابرابر ppm 095/0 و میانگین ماهیانه غلظت ذرات قابل ته نشینی 35/ 13بوده که در تمامی موارد پائین تر از حدود مجاز بوده است. ضمنا شاخص کیفیت هوا نیز در تمامی موارد نمونه برداری در حد استانداردهای کیفیت هوای ایران، رهنمودهای WHO واستانداردهای آژانس حفاظت محیط زیست آمریکا است.   نتیجه گیری: کیفیت بهداشتی هوای پیرامون مجتمع در وضعیت خوبی از نظر حفظ سلامتی کارکنان و مراجعین بر خوردار است. با توجه به اینکه غلظت ذرات معلق ( PM10 ) و SO2 از سایر آلاینده ها بیشتر بوده است، بایستی از هم اکنون تمهیدات لازم جهت جلوگیری از بالا رفتن غلظت آنها که ممکن است در آینده با همین روند از استاندارد ها تجاوز نماید، اتخاذ نمود.

Title: بررسی میزان تاثیر تئاتر بر روی آگاهی، نگرش و عملکرد زنان مراجعه‌کننده به مراکز بهداشتی درمانی شهر یزد در زمینه مصرف روغن و چربی

http://tbj.ssu.ac.ir/article-1-556-fa.html

مقدمه: مطالعه و ارتقاء آگاهی، نگرش و عملکرد خانوارها به خصوص زنان در زمینه مصرف روغن و چربی، در پیش‌بینی و ارتقاء وضعیت سلامت جامعه از جایگاه مهمی برخوردار است و تئاتر به عنوان یک روش آموزشی می‌توان جهت افزایش آگاهی، تغییر نگرش و بهبود عملکرد موثر واقع گردد. هدف از این مطالعه بررسی میزان تاثیر تئاتر بر روی آگاهی، نگرش و عملکرد زنان مراجعه‌کننده به مراکز بهداشتی درمانی شهر یزد در زمینه مصرف روغن و چربی بوده است .   روش‌ بررسی: این مطالعه تجربی از نوع قبل و بعد بوده که تعداد 130 نفر از زنان که به روش تصادفی خوشه‌ای از سه مرکز بهداشتی درمانی شهری آزادشهر، نیکوپور و رحمت‌آباد انتخاب و در دو گروه مورد و شاهد به طور تصادفی و مساوی تقسیم که 65 نفر در گروه مورد و 65 نفر در گروه شاهد قرار گرفتند. ابزار جمع‌آوری داده‌ها پرسشنامه‌ای مشتمل بر متغیرهای دموگرافیک به انضمام سوالات آگاهی، نگرش و عملکرد بود. پایایی و روایی پرسشنامه مورد سنجش قرار گرفت و تایید گردید. ابتدا پرسشنامه برای دو گروه تکمیل سپس تئاتر برای گروه مورد اجرا و یک ماه پس از اجرا مجدداً همان پرسشنامه برای گروه مورد و شاهد اجرا و تاثیر تئاتر مورد ارزیابی قرار گرفت. داده‌ها با استفاده از نرم‌افزار16 SPSS. و به کارگیری آمار توصیفی و پارامتریک و نان پارامتریک مورد تجزیه و تحلیل قرار گرفت.   یافته ها: میانگین نمره آگاهی گروه مورد قبل از اجرای تئاتر از 26 نمره 53/19 بود که با اجرای تئاتر به 38/21 رسید (001/0 P< ). میانگین نمره نگرش گروه مورد قبل از اجرای تئاتر از 40 نمره 13/31 بود که با اجرای تئاتر به 8/32 رسید (001/0 P< ) و میانگین نمره عملکرد گروه مورد قبل از اجرای تئاتر از 24 نمره 13/13 بود که بعد از اجرای تئاتر به 70/15 رسید (001/0 P< ) که بین میانگین نمره آگاهی، نگرش و عملکرد قبل و بعد از اجرای تئاتر اختلاف معنی‌داری بوده است.همچنین نتیجه آزمون T-Paired حاکی است که در گروه شاهد بین نمره آگاهی قبل و بعد اختلاف معنی‌داری وجود دارد ((01/0 P< )ولی بین نمرات نگرش و عملکرد و قبل و بعد اختلاف معنی‌داری وجود ندارد.   نتیجه‌گیری: نتایج این مطالعه حاکی از آن بود که اجرای تئاتر باعث ارتقاء آگاهی، تغییر نگرش و بهبود عملکرد زنان گروه مورد پیرامون مصرف روغن و چربی گردیده است. لذا می‌توان از تئاتر به عنوان یک روش آموزشی جهت آموزش موضوعات مختلف در زمینه سلامت استفاده نمود و همچنین اجرا و استمرار این برنامه‌ها باید مورد توجه سیستم بهداشتی باشد.

Title: بررسی تأثیرگذاری اسلوب ارتباطی بر پیامدهای کاری پرسنل درمانی با تاکید بر نقش رضایتمندی ارتباطی

http://tbj.ssu.ac.ir/article-1-557-fa.html

مقدمه: ارتباطات سازمانی از فعالیت­های اصلی مدیریت و کارکنان سازمان به ویژه مشاغل درمانی محسوب می­شود. مدیران بیمارستان­ها به منظور ارتقاء پیامدهای کاری باید اثرات اسلوب ارتباطی بر این پیامدها را شناسایی نمایند. رفتار شهروند سازمانی، تعهد سازمانی و رضایتمندی شغلی به عنوان مهمترین پیامدهای کاری کارکنان مطرح شده­اند. این مطالعه با هدف تعیین رابطه اسلوب ارتباطی پرستاران با رضایتمندی ارتباطی درک شده و پیامدهای کاری پرستاران و ارائه مدلی مفهومی انجام شده است.   روش بررسی: در این مطالعه کاربردی از استراتژی تحقیق پیمایشی به منظور گردآوری داده استفاده شده است. جامعه آماری این مطالعه متشکل از پرسنل درمانی بیمارستان قائم(عج) شهر مشهد بود که 268 نفر به عنوان نمونه تصادفی طبقه­ای در تحقیق مشارکت داشته­اند. داده­های مطالعه از طریق پرسشنامه­های استاندارد بدست آمدند. اعتبار ابزارها به کمک تحلیل عاملی و پایایی آنها با کمک آلفای کرونباخ تائید گردید. داده­ها با استفاده از نرم افزار smart pls و بکارگیری روشهای آماری تجزیه و تحلیل گردید.   یافته­ها: نتایج نشان داد اسلوب ارتباطی پرستاران با ضرایب رگرسیونی 562/0، 216/0، 218/0 و 204/0 به ترتیب با رضایتمندی ارتباطی درک شده، رضایتمندی شغلی، رفتار شهروند سازمانی و تعهد سازمانی پرستاران و همچنین رضایتمندی ارتباطی با ضرایب رگرسیونی 455/0، 316/0 و 397/0 با رضایتمندی شغلی، رفتار شهروند سازمانی و تعهد سازمانی پرستاران رابطه معناداری دارد. همچنین نقش واسط رضایتمندی ارتباطی در ارتباط بین اسلوب ارتباطی پرستاران و پیامدهای کاری پرستاران به دلیل بزرگتر بودن رابطه غیر مستقیم از رابطه مستقیم تائید شد.   نتیجه­گیری: باتوجه به آن­که از نظر جامعه مورد بررسی، اسلوب ارتباطی به میزان زیادی بر پیامدهای کاری و روانی پرستاران تاثیر دارند. بنابراین، اثرات این تحقیق برای مدیران بیمارستان­هابه منظور گسترش رفتارهای بهره­ور مفید است. زیرا توفیق در اجرای اسلوب ارتباطی، خود مهم­ترین و نخستین گام برای بهره­ور نمودن پرستاران می­باشد.

Title: بررسی میزان آلودگی قارچی سطوح استخرهای سرپوشیده شهر یزد در سال 1390

http://tbj.ssu.ac.ir/article-1-558-fa.html

مقدمه : مطالعات نشان می­دهد که شنا کردن در استخرهای شنا می­تواند باعث انتقال عفونت­ها شود. بنابراین هنوز این نگرانی وجود دارد که ممکن است شناگران به وسیله تماس با آب و محیط اطراف استخر به بیماریهای قارچی دچار شوند. لذا این تحقیق با هدف تعیین میزان آلودگی های قارچی در استخرهای سرپوشیده شهر یزد در سال 1390 انجام گرفت.   روش بررسی: در این مطالعه توصیفی، به صورت سرشماری تمامی استخرهای شنای سرپوشیده شهر یزد (12 استخر) در طول بهار و تابستان سال 1390 و ماهی 2 بار از نظر آلودگی قارچی مورد بررسی قرار گرفتند. برای تعیین آلودگی های قارچی از روش نمونه گیری با موکت استریل استفاده شد. 60 نمونه از سطوح محیطی مختلف هر استخر مورد بررسی (دوش، سطح رختکن، صندلی و محل نشستن در سونا، حمام، سکوها، اطراف استخر) و بطور متوسط از 5 ناحیه هر استخر و در مجموع 720 نمونه از تمام استخرها برای جداسازی آلودگی قارچی بخصوص در روزهای جمعه که بیشترین مراجعه کننده داشتند مورد آزمایش قرار گرفتند.    یافته ها: یافته ها نشان داد که از مجموع 720 نمونه کشت شده در این تحقیق مجموعآ تعداد 495 نمونه (7/68%درصد) دارای یک یا چند آلودگی قارچی بودند. آلودگیهای قارچی جدا شده شامل قارچهای ساپروفیت کپکی 443 نمونه (5/89%)، مخمری ساپروفیتی61 نمونه (7/9%) و قارچهای بیماریزای درماتوفیتی 4 نمونه (8/0%) بود که با بررسی آماری توصیفی بیشتر از نواحی دوشها، رختکن، سونا و کناره های استخر جدا شدند.    نتیجه گیری: با توجه به نتایج حاصله از این مطالعه بخصوص جدا شدن قارچهای درماتوفیت، لازم است که به بهداشت و گندزدایی بهتر جهت کاهش آلودگی آب و محیط اطراف استخرها توجه و نظارت بیشتری صورت گیرد.      این مقاله حاصل از پایان نامه دانشجوی کارشناسی ارشد دانشگاه علوم پزشکی شهید صدوقی می باشد.

Title: بررسی عوامل محیطی موثر بر بروز اسهال در شهرستان تنگستان استان بوشهر در سال 1390

http://tbj.ssu.ac.ir/article-1-560-fa.html

مقدمه: اپیدمی اسهال هر ساله در اکثر نقاط جهان به ویژه کشورهای در حال توسعه اتفاق می افتد. این مطالعه با هدف بررسی عوامل محیطی موثر بر بروز اسهال در شهرستان تنگستان استان بوشهر در سال 1390 انجام شد.   روش بررسی: این مطالعه از نوع مورد – شاهد بود. داده بوسیله چک لیست جمع آوری گردید. 262 نفر در گروه مورد که دارای بیماری اسهال بودند و 262 نفر در گروه شاهد چک لیست را تکمیل کردند. پس از تکمیل و جمع آوری چک لیست ها برای آنالیز به نرم افزار آماری spss وارد شدند و بوسیله آمار توصیفی و رگرسیون لجستیک مورد تجزیه و تحلیل قرار گرفتند.   یافته ها: میانگین سنی شرکت کنندگان 71/24 سال ، منبع آب آشامیدنی 7/17 درصد آب انبار بود، 4/63 درصد در مناطق روستایی ساکن بودند. 8/66 درصد از شرکت کنندگان سبزی مصرفی را فقط با آب شستشو داده بودند،6/99درصد فاضلاب آنها بصورت بهداشتی دفع می شد و 9/6 درصد توالت غیر بهداشتی داشتند. رگرسیون لجستیک نشان داد نوع مخزن آب شرب، نوع غذا و سبزی مصرفی و وجود حشرات ناقل در محل زندگی به ترتیب 20/1 ،93/2 و 18/2 و 35/2 برابر شانس ابتلا به بیماری اسهال را در گروه مورد نسبت به گروه شاهد بیشتر می کنند.   نتیجه گیری: با توجه به نقش تعیین کننده محیط زیست در بروز بیماری اسهال بخصوص در فصول گرم سال دادن آموزش های بهداشتی به مردم مخصوصا کسانی که در زمینه تهیه و توزیع مواد غذایی نقش دارند همچنین کلر زنی آب انبار ها می تواند در کنترل بیماری اسهال کمک کننده باشد.

Title: طراحی و ساخت محفظه تولید و شبیه سازی آلاینده های هوابرد

http://tbj.ssu.ac.ir/article-1-562-fa.html

مقدمه: افزایش توانمندی و مهارت عملی دانشجویان با استفاده از روشهای نوین آموزشی جهت بررسی و اندازه‌گیری دقیق آن‌ها ضروری می باشد از طرفی محدودیت‌های موجود جهت حضور دانشجویان در صنعت و همچنین امکان مواجهه آنها با خطرات محیط کار، ضرورت این امر را مضاعف می نماید لذا این تحقیق با هدف طراحی و ساخت دستگاهی با قابلیت تولید انواع آلاینده های ذره‌ای، گازها و بخارات در محیط آزمایشگاه جهت تسهیل در آموزش عملی دانشجویان و همچنین انجام تحقیقات انجام شد.   روش بررسی: در این بررسی، با توجه به بازنگری طراحی های انجام شده قبلی در دنیا در خصوص تجهیزات موجود در زمینه شبیه سازی آلاینده های هوابرد و همچنین نظرسنجی از متخصصین بهداشت حرفه ای خصوصیات دستگاه مشخص و طراحی و ساخت آن بویژه قابلیت دستگاه جهت انتشار ذرات هوابرد ، نمونه برداری و اندازه گیری آن به صورت ابداعی انجام گرفت همچنین سادگی، مقرون به صرفه بودن، قابلیت حمل و قابل شستشو بودن از دیگر ویژگی های این دستگاه می باشد   یافته‌ها: دستگاه ساخته شده متشکل از محفظه اصلی، محفظه گرمایش، مجموعه دمنده دمپردار و جعبه کنترل قابلیت تولید و شبیه سازی انواع آلاینده‌های ذره‌ای و گازی شکل را داشته و با ساخت این دستگاه امکان نمونه‌برداری انواع آلاینده‌ها در داخل آزمایشگاه فراهم گردید. نتایج حاصل از ارزیابی عملکرد دستگاه نشان داد که غلظت ذرات قابل استنشاق پس از رها سازی در شرایط یکسان تقریبا یکنواخت می باشد.   نتیجه‌گیری: دستگاه ساخته شده پس از آزمایش در اداره ثبت اختراعات کشور ثبت شده (به شماره 72272) و با توجه به نتایج قابل قبول آن در چند ترم متوالی جهت آموزش عملی دانشجویان این دانشگاه استفاده گردید.لذا می‌توان به عنوان یک وسیله کمک آموزشی جهت آزمایشگاه بهداشت حرفه ای دانشگاه ها مورد استفاده قرار گیرد.

Title: بررسی رابطه خوشبینی با سلامت عمومی در دانشجویان دانشگاههای شهر یزد

http://tbj.ssu.ac.ir/article-1-563-fa.html

مقدمه: سازمان جهانی بهداشت سلامت عمومی را سلامتی کامل جسمی، اجتماعی و روانی نه فقط فقدان بیماری یا ناتوانی تعریف می کند. خوشبینی هم بر روی سلامت روانی و هم جسمانی تاثیر دارد. لذا این مطالعه با هدف بررسی ارتباط خوشبینی با سلامت عمومی در دانشجویان دانشگاههای شهر یزد طراحی و اجرا شد.   روش بررسی: مطالعه حاضر توصیفی- همبستگی می باشد جامعه مورد بررسی 300 نفر از دانشجویان دانشگاههای علوم پزشکی، یزد و آزاد اسلامی شهر یزد بوده که به روش طبقه ای تصادفی انتخاب شدند. ابزار جمع آوری داده ها پرسشنامه های استاندارد سلامت عمومی( GHQ-28 ) و خوشبینی( LOT ) بوده که روایی و پایایی آن در مطالعات قبلی مورد تایید قرار گرفت. پس از تکمیل پرسشنامه ها، داده ها با استفاده از نرم افزار آماری SPSS نسخه 16 و آزمون های آماری ANOVA ، T-test و همبستگی پیرسون مورد تجزیه و تحلیل قرار گرفت.   یافته ها: خوشبینی با سلامت عمومی (001/0 P< و 472/0 r= )، علائم جسمانی(001/0 P< و 800/0 r= )، اضطراب و اختلال خواب(001/0 P< و 849/0 r= )، اختلال در کارکرد اجتماعی(001/0 P< و 705/0 r= ) و افسردگی(001/0 P< و 860/0 r= ) همبستگی قوی داشت. اختلاف معنی داری بین میانگین نمره سلامت عمومی و خوشبینی دانشجویان دانشگاههای مختلف مشاهده گردید(به ترتیب 001/0 P= و 045/0 P= )، بطوری که دانشجویان دانشگاه علوم پزشکی شهید صدوقی دارای میانگین نمره سلامت عمومی و خوشبینی پایین تری نسبت به دو دانشگاه دیگر یعنی آزاد اسلامی و یزد بودند.   نتیجه گیری: با توجه به نتایج این مطالعه خوشبینی وسلامت عمومی رابطه بسیارقوی ونزدیکی بایکدیگر دارندلذا لازم است کوشش شود خوشبینی دانشجویان به هرطریق ممکن افزایش یابد تاسلامت عمومی آن ها نیز افزایش یابد.    مقاله حاضر برگرفته از پایان نامه تحصیلی مقطع کارشناسی ارشد دانشگاه علوم پزشکی و خدمات بهداشتی درمانی شهید صدوقی یزد می باشد.

Title: بررسی عوامل موثر بر آلودگی به شپش سر، در بین دانش آموزان دختر مدارس ابتدایی ارجاع داده شده به مرکز بهداشت شهرستان رباط کریم سال تحصیلی 88-87

http://tbj.ssu.ac.ir/article-1-564-fa.html

مقدمه : شپش، انگل خارجی و دائمی انسان می باشد، که موجب تحریک، خارش ، خستگی، ضعف عمومی و اختلال در یادگیری می گردد. پژوهش حاضر با هدف تعیین عوامل موثر بر آلودگی به شپش سر، در بین دانش آموزان دختر مدارس ابتدایی شهرستان رباط کریم طی سال تحصیلی 88-87طراحی و اجراگردید.   روش بررسی : این مطالعه با روش نمونه گیری چند مرحله ای خوشه ای به روش توصیفی انجام پذیرفت. جامعه تحت مطالعه دانش آموزان دختر ابتدایی شهرستان رباط کریم بود، حجم نمونه مورد بررسی 10000نفر محاسبه گردید. روش گرد آوری اطلاعات مشاهده ای و تکمیل پرسشنامه بوده، و تجزیه و تحلیل داده ها با استفاده از نرم افزار SPSS و تست X2 انجام شد.   متغیر های مورد بررسی شامل تعداد اعضای خانواده، تعداد اتاق، تحصیلات وشغل والدین، ، دسترسی به آب سالم، وجود حمام در منزل، تعداد استحمام در هفته و مواد شوینده، می باشند.   یافته ها: از مجموع 10000نمونه، 125 نفر( 25/1 درصد) دارای شپش سر بودند که از بین آنها بیشترین درصد مربوط به دانش آموزان 7ساله بود. در این مطالعه ارتباط معنی داری بین میزان آلودگی و عوامل مستعد کننده ای چون دسترسی به آب سالم، مواد شوینده، وجود حمام و تعداد استحمام مشاهده نگردید.   نتیجه گیری :با توجه به نتایج بدست آمده چنین بنظر می رسد که مهمترین اقدام انجام شده در 6/81درصدموارد مراجعه مبتلایان به مراکز بهداشتی درمانی بود که پس از آن بازرسی دوره ای نشان دهنده عدم وجود شپش در مبتلایان بود. همچنین بر طبق مطالعات انجام شده بالا بودن سطح سواد سبب کاهش ابتلا می گردد. بنابراین آموزش مادران و پیگیری وضعیت درمان سبب کنترل میزان شیوع آلودگی به شپش می گردد.

Title: بررسی تأثیر آموزش بر آگاهی، نگرش و عملکرد دانش آموزان پایه دوم دبیرستان های پسرانه شهر زاهدان در خصوص بیماری ایدز در سال 1390

http://tbj.ssu.ac.ir/article-1-565-fa.html

مقدمه: با توجه به اهمیت جوانان در گسترش و خطر ابتلا به ایدز و ا ز آنجا که تنها ابزار کنترل بیماری، آموزش می باشد، این مطالعه به منظور ارزیابی تأثیر آموزش بر آگاهی،نگرش و عملکرد دانش آموزان پسر پایه دوم دبیرستانهای شهر زاهدان در ارتباط با ایدز طراحی واجرا گردید.   روش بررسی: این مطالعه تجربی از نوع قبل و بعد، با روش نمونه گیری چند مرحله ای تصادفی بر روی 280 دانش آموز پسر پایه دوم دبیرستانهای شهر زاهدان انجام شد. اطلاعات با یک پرسشنامه چهار قسمتی در دو مرحله قبل و یک ماه بعد از مداخله تکمیل شد. داده ها با استفاده از نرم افزار SPSS در سطح معنی داری05/0= α آنالیز شدند.   یافته ها: میانگین نمره آگاهی بعد از آموزش از08/3±80/11 به97/1±32/16(07/0 P= )، نمره نگرش از02/3 ± 17/22 به 23/3±57/27 (02/0 P= ) ونمره عملکرداز14/1 ± 4به33/1 ± 63/5 افزایش یافت(06/0 P= ). آگاهی27% دانش آموزان قبل از مداخله ضعیف، 40%متوسط و33%خوب بودکه بعد از مداخله به 4/5% ضعیف،6/20%متوسط و 74% خوب افزایش یافت.نگرش 2/20% دانش آموزان قبل از مداخله ضعیف، 8/51%متوسط و 28%خوب بود که بعد از مداخله به 7/. % ضعیف 9/7%متوسط و 4/91% خوب تغییر یافت. همچنین عملکرد دانش آموزان قبل از مداخله 1/38%ضعیف، 8/51%متوسط و1/10%خوب بود که بعد ازمداخله به 7/.% ضعیف، 7/15% متوسط و 6/83% به خوب تغییر یافت.   نتیجه گیری: از آنجا که تأثیر آموزش بر ارتقاء سطح آگاهی ، نگرش و عملکرد دانش آموزان تایید گردید، اجرای برنامه های آموزش بهداشت به عنوان یک اولویت آموزشی در مدارس می بایست مورد توجه مسئولین و مدیران بهداشتی و آموزشی کشور قرار گیرد.

Title: بررسی وضعیت بهداشتی کشتارگاه‌های دام وطیور استان یزد

http://tbj.ssu.ac.ir/article-1-566-fa.html

مقدمه: کشتارگاه به عنوان محلی جهت تأمین منبع غذایی اساسی برای انسانها، نقش مهمی را در سلامت اجتماع ایفا می کند. در صورتی که اصول بهداشتی درکشتارگاهها رعایت نشود، افراد مصرف کننده گوشت ممکن است به بیماریهای مختلف مبتلا شوند. باتوجه به اهمیت موضوع، مطالعه حاضر با هدف تعیین وضعیت بهداشتی کشتارگاه های استان یزد انجام شد.   روش بررسی: دراین مطالعه توصیفی- تحلیلی،14 کشتارگاه استان یزد به روش سرشماری موردبررسی قرارگرفتند. باتوجه به اهداف تحقیق، پرسشنامه ای جهت بررسی وضعیت بهداشتی کشتارگاههای دام و طیور تهیه شد. برای پاسخ دادن به هرسؤال سه گزینه شامل"مطلوب "،" نامطلوب " و"متوسط" درنظرگرفته شده بود. سپس دو نفراز کارشناسان بهداشت محیط طی 4 جلسه آموزش های لازم در خصوص چگونگی امتیازدهی به هرکدام ازموارد را فراگرفتند. بر حسب اهمیت موضوع به هرکدام ازآیتم هاباحضور کارشناسان بهداشت محیط وبه صورت کارگروهی و مشاوره ای امتیاز لازم داده شد.   یافته ها: وضعیت کشتارگاه های مورد بررسی ازنظر بهداشت فردی(7%)، امکانات موردنیاز (43%)، ساختمان(64.5%)، ابزارووسایل کار(57%)، دفع فاضلاب(14%)، آب(93%) و جمع آوری ودفع زباله(57%) مطلوب بوده است.   نتیجه گیری: بطورکلی این مطالعه نشان داد که فقط کشتارگاه جدید یزد دارای استانداردهای ضروری بهداشت محیطی بوده وسایرکشتارگاه ها دارای نواقص بهداشتی متعددی هستندکه این مسأله توجه ودقت بیشتری را درجهت بهبود و ارتقای وضعیت بهداشتی کشتارگاهها ازسوی مسئولین ذیربط را می طلبد.

Title: بررسی حذف مواد آلی در نیزار مصنوعی زیر سطحی : تعیین ضرایب سینتیکی

http://tbj.ssu.ac.ir/article-1-567-fa.html

مقدمه: در بررسی سیستم های ­ طبیعی تصفیه فاضلاب به کمک نیزار مصنوعی، مدل های علمی و عملی از جنبه های فیزیکی، هیدرولیکی و فرایندی در مقیاس واقعی ­ و ­ تعیین قوت و ضعف این سیستم ­­ ها، می ­ تواند نقشی مهمی جهت احداث سایر واحدهای مشابه ایفا نماید. هدف اولیه طراحی سیستم بسترهای نی ­ در تصفیه فاضلاب، حذف مواد آلی می باشد، لذا تعیین اولیه سینتیک های حذف BOD 5 و COD جریان فاضلاب ضروری است.   روش ­ بررسی: پژوهش حاضر توصیفی- مقطعی بوده و سینتیک واکنش­های بیولوژیکی حذف مواد آلی در بسترهای نی را مورد بررسی قرار می دهد. بدین­منظور، نمونه­ها در سه محل از تالاب به­­طور ماهیانه طی شش ماه برداشت شدندو مقدار حذف مواد آلی با سنجش s COD و s BOD 5 انجام­گرفت. سپس نتایج با مدلهای سینتیک مرتبه اول ، مرتبه دوم و استوورکین­کانن و پارامترهای آماری تجزیه و تحلیل شدند.   یافته ­ ها: نتایج، متوسط غلظت خروجی s COD و s BOD 5 از تالاب شاهد و دارای نی ­ را به ترتیب 49/101، 92/56 و 78/81 ، 18/46 میلی ­ گرم ­ بر لیتر نشان داد. در­خصوص پیش­بینی رفتار حذف مواد آلی، مدل استوورکین کانن نزدیک­ترین ارتباط بین پیش بینی های تئوری و ­ داده های میدانی ارائه نمود و مقدار ضریب حداکثر مصرف ماده آلی در مورد s COD و ­ s BOD 5 در تالاب دارای نی 48/120 و 125 و در شاهد 3/33 و 5/38 میلی گرم در لیتر در روز بدست آمد .   نتیجه ­ گیری: با توجه به نتایج، تصفیه­خانه فاضلاب یزد بدلیل شرایط مناسب جوی و طراحی، درصد حذف مواد آلی آن زیاد بوده که موجب کاهش چشمگیر آلودگی شده و راندمان کافی جهت برآوردن استانداردهای خروجی پساب را فراهم می­آورد .

Title: بررسی سطح فعالیت جسمانی و عوامل موثر برآن در دانش آموزان پسر دوره راهنمائی یک شهرک مسکونی در تهران

http://tbj.ssu.ac.ir/article-1-570-fa.html

مقدمه :فعالیت جسمانی منظم و کافی درسنین نوجوانی به عنوان یک عامل بسیار مهم و تعیین کننده سلامت دربزرگسالی شناخته شده است. هدف مطالعه، تعیین سطح فعالیت جسمانی و عوامل موثر برآن دردانش آموزان پسر دوره راهنمائی دریک شهرک مسکونی در تهران بود.   روش بررسی : این مطالعه توصیفی مقطعی برروی  کلیه دانش آموزان (179نفردانش‌آموز پسر) انجام گردید. اطلاعات با استفاده از پرسشنامه محقق ساخته و درحضور پرسشگرجمع‌آوری گردید.برای روایی پرسشنامه از روش اعتبار محتوا و پایایی آن از روش آلفای کرونباخ ( 76%) استفاده شد.اطلاعات جمع‌آوری شده با استفاده ازآمار توصیفی و آزمونهای کای اسکوئر وآنالیز واریانس و بانرم افزار 15 SPSS تجزیه و تحلیل گردید.درنهایت بر اساس نتایج طرح،راهکارهای مناسب برای ارتقاء سطح فعالیت جسمانی دانش آموزان ارائه شد.   یافته‌ها : سطح فعالیت جسمانی دانش آموزان به ترتیب 3/36 درصد کم، 1/34 درصد متوسط و 6/29 درصد شدید بودند. جامعه مورد مطالعه میزان دسترسی به امکانات ورزشی در منزل را به ترتیب2/54 درصد کم، 4/27 درصد متوسط و 4/18 درصد زیاد و در مدرسه به ترتیب 3/55 درصد کم،43درصد متوسط و7/1درصد زیاد ارزیابی نموده اند.3/74درصد دانش­آموزان از حمایت کم و7/25 درصد از حمایت خوب خانواده برخورداربودند. سطح فعالیت جسمانی دانش­آموزان با متغیرهای BMI ،پایه کلاس و سن ارتباط معنی دار و با متغیرهای سطح تحصیلات و شغل والدین ارتباط معنی داری نداشت.   نتیجه گیری: برای افزایش فعالیت جسمانی در بین دانش آموزان ، استفاده از مداخلات مدرسه­ محور و افزایش حمایت خانواده ها ضروری است. همچنین بایستی امکانات ورزشی درمنزل ومدرسه ایجاد و دسترسی به آن را تسهیل نمود و فواید فعالیت جسمانی مداوم را به دانش­آموزان و خانواده­ها آموزش داد.   

Title: بررسی رفتارهای مدیریت غذا در زنان ساکن شهر کرمان در سال1390

http://tbj.ssu.ac.ir/article-1-569-fa.html

چکیده مقدمه: بیماری های ناشی ازغذا درنتیجه ورود پاتوژن های میکروبی به زنجیره غذایی ایجاد می شوند. مدیریت ناسالم غذا عامل اصلی برای انتقال بیماریهای ناشی ازغذا می باشند. نظر به اهمیت موضوع و محدودیت مطالعات در ایران، مطالعه حاضر با هدف بررسی رفتار های مدیریت غذا در زنان ساکن شهر کرمان انجام شد. روش بررسی:این مطالعه توصیفی تحلیلی به روش مقطعی بر روی 320 نفر از زنان ساکن شهرکرمان که مسئولیت اداره و آماده سازی غذا در منزل به عهده آنها بود، انجام شد. ابزارگردآوری داده ها پرسشنامه بود که روایی آن توسط نظرات خبرگان، و پایایی آن بوسیله آزمون آلفای کرونباخ مورد تأیید قرار گرفت. پرسشنامه شامل سؤالات ویژگی های فردی و سؤالات مربوط به رفتارهای مدیریت غذا، در چهار بخش بهداشت، جداسازی، پختن و سرد کردن مواد غذایی بود. جهت تجزیه و تحلیل داده ها از آزمون های ANOVA و T-Test استفاده شد. یافته ها: در این مطالعه 52% افراد همیشه غذای اصلی را در منزل تهیه می کردند. 4/44%کمتر از یک وعده در هفته غذا را در بیرون از منزل مصرف می کردند. شرکت کنندگان به ترتیب، در بخش بهداشت از مجموع 40 نمره، میانگین 94/2±52/34 نمره، در بخش جداسازی از 36 نمره، میانگین97/2± 99/30 نمره، در بخش پختن از مجموع 32 نمره، میانگین54/2 ±25/26 نمره و دربخش سرد کردن ازمجموع 56 نمره، میانگین 88/2 ±78/37 نمره بدست آوردند. در بخش بهداشت، شستن دستها بلافاصله بعد از تماس با گوشت، مرغ و ماهی خام و عدم مصرف میوه های شسته نشده، بیشترین رفتار بهداشتی گزارش شده بود. بیشترین رفتار غیر بهداشتی گزارش شده نیز"مزه کردن غذا برای اطمینان از فاسد نبودن آن" مشخص شد. در بخش جداسازی بیشترین رفتار غلط گزارش شده "گذاشتن گوشت خام پایین تر از غذاهای پخته در یخچال" بود. در بخش پختن مواد غذایی، عدم مصرف شیر محلی نجوشیده بیشترین رفتار بهداشتی گزارش شده بود و در این بخش" 20 دقیقه جوشاندن قوطی کنسرو قبل از مصرف" کمترین رفتار گزارش شده بود. بیشترین رفتار غلط انجام شده در بخش سرد کردن مربوط به سرد کردن غذاهای پخته شده قبل از گذاشتن در یخچال در دمای اتاق بود. بین میانگین نمره کلی رفتارمدیریت غذا وزیرمقیاسهای آن با متغیرهای دموگرافیک و نیز سایر متغیرهای زمینه ای ارتباط معنا دار آماری مشاهده نشد. نتیجه گیری: رفتار های شرکت کنندگان در زمینه مدیریت غذا بویژه به لحاظ سرد کردن مواد غذایی در حد ایده آل نیست. نتایج نشان دهنده این است که تعداد زیادی از افراد تاکنون با این گونه مفاهیم روبرو نشده بودند. سطح پایین بعضی رفتارها دلیل محکمی بر عدم اگاهی افراد است. بنابراین برنامه های آموزشی بایستی با توجه به نقاط قوت و ضعف افراد در هربخش ارائه گردد. این مقاله حاصل از پایان نامه دانشجوی کارشناسی ارشد دانشگاه علوم پزشکی شهید صدوقی یزد می باشد.

Title: تاثیر برنامه آموزشی مبتنی بر الگوی پرسید بر رفتارهای ایمنی دانش آموزان پسرمدارس ابتدایی شهر اهواز در برابر وقوع زلزله

http://tbj.ssu.ac.ir/article-1-571-fa.html

چکیده مقدمه: براساس گزارش های سازمان ملل متحد، سازمان جهانی بلایا، کشور ایران نه تنها در زمره کشورهای بلاخیز جهان رتبه بندی شده است، بلکه رتبه نخست را از نظر بیشترین تعداد موارد مرگ متعاقب بروز زلزله داشته است.این مطالعه با هدف تاثیر برنامه آموزشی الگوی پرسید، پروسید بر رفتارهای ایمنی دانش آموزان مدارس ابتدایی شهر اهواز در برابر وقوع زلزله طراحی شده است. روش بررسی: این پژوهش یک مطالعه مورد شاهدی است که جهت بررسی رفتارهای ایمنی دانش آموزان در سال 1390 انجام شد. 400 نفر از دانش آموزان به روش نمونه گیری خوشه ای و تصادفی ساده از بین دو دبستان شهر اهواز انتخاب شدند. اطلاعات با استفاده از پرسشنامه و چک لیست، جمع آوری شد. پرسشنامه حاوی سوالاتی در بخش های مشخصات دموگرافیک، آگاهی، نگرش و رفتار عوامل قادر کننده و عوامل تقویت کننده بود. برای تجزیه و تحلیل داده ها از نرم افزارSPSS ، آزمونهای t مستقل و زوج و آزمون کای دو استفاده شد. یافته ها:پس از مداخله آموزشی در گروه مورد نسبت به گروه شاهد میانگین نمره آگاهی و میانگین نمره نگرش در خصوص رعایت نکات ایمنی در برابر وقوع زلزله (عوامل مستعدکننده ) (001/0P<)،استفاده از منابع آموزشی،تشکیل کلاس آموزشی و شرکت در کلاس آموزشی (عوامل قادرکننده ) (001/0P<)و تشویق خانواده و معلمین (عوامل تقویت کننده)و رفتارهای ایمنی در هنگام وقوع زلزله افزایش معنی دار یافته اند(001/0P<). نتیجه گیری: نتایج فوق نشان دهنده تاثیر مثبت برنامه مداخله آموزشی بر اساس الگوی پرسید و اجزای اصلی آن (عوامل مستعدکننده،قادرکننده و تقویت کننده) در افزایش رفتارهای ایمنی در هنگام وقوع زلزله در جامعه مورد مطالعه می باشد. واژه های کلیدی: آموزش بهداشت، بهداشت مدارس،الگوی پرسید، رفتارهای ایمنی

Title: اجرای برنامه ادغام یافته کودک سالم و شاخص های کودکان درشبکه بهداشت و درمان شهرستان نور

http://tbj.ssu.ac.ir/article-1-572-fa.html

مقدمه: تولد سالانه بیش از یک میلیون کودک درکشور ما دلیلی بسیار محکم برای پرداختن به جنبه های جسمی و روانی تامین سلامت کودک است. این پژوهش با هدف بررسی ارتباط بین اجرای برنامه ادغام یافته کودک سالم باشاخص های کودکان در شبکه بهداشت و درمان شهرستان نور به منظور اصلاح برنامه و کمک به بهبود سلامت کودکان انجام پذیرفت.   روش‌بررسی : پژوهش‌ حاضر مقطعی می باشد که در فاصله زمانی سال های 1386 تا 1390 به جزسال 1388که سال اجرای برنامه بود، انجام پذیرفت. آمار مرتبط با برنامه ادغام یافته کودک سالم در شبکه بهداشت و درمان شهرستان نور گرد‌آوری شد . ابزار گردآوری داده‌ها فرم جمع‌آوری اطلاعات ساخته پژوهشگر بود. جهت استخراج نتایج پژوهش از آزمون کلموگروف اسمیرنوف، ضریب همبستگی پیرسون و آزمون تی زوجی استفاده شد .   یافته‌ها : بین اجرای برنامه ادغام یافته کودک سالم و میزان مرگ ومیرکودکان زیر یکسال و زیر 5 سال و میزان پوشش واکسیناسیون رابطه معنی داری مشاهده نشد، اما در شاخص میزان پنومونی درکودکان زیر 5سال و میزان ارجاع کودکان به پزشک و میزان اختلال رشد کودکان و میزان حوادث، مسمومیت وسوختگی ها، رابطه معنی داری درشبکه بهداشت و درمان شهرستان نور مشاهده گردید.   نتیجه‌گیری: نتایج حاصله به روشنی بیانگر تاثیرمثبت اجرای برنامه ادغام یافته کودک سالم بربهبود شاخصهای اختلال رشدکودکان، میزان پنومونی و میزان ارجاع کودکان به پزشک ومیزان حوادث، مسمومیت وسوختگی ها درکودکان می‌باشد ولی باتوجه به نقاط ضعف شناسایی شده، با مداخلات موثر و برنامه ریزی بهترتوسط مدیران می‌توان گامی در جهت بهبود برنامه و ارتقاء شاخصهای بهداشتی و ارتقاء سلامت جامعه برداشت.
